# *H3* K27M mutation in rosette-forming glioneuronal tumors: a potential diagnostic pitfall

**DOI:** 10.1007/s00428-024-03739-2

**Published:** 2024-01-17

**Authors:** Elena Marastoni, Serena Ammendola, Sabrina Rossi, Isabella Giovannoni, Giuseppe Broggi, Barbara Masotto, Alberto Feletti, Valeria Barresi

**Affiliations:** 1 Department of Diagnostics and Public Health, University of Verona, Policlinico G.B. Rossi, P.le L.A. Scuro, 10, 37138 Verona, Italy; 2 Unit of Anatomic Pathology, Ospedale Pediatrico Bambino Gesù, Rome, Italy; 3 Department of Medical and Surgical Sciences and Advanced Technologies, G.F. Ingrassia, Anatomic Pathology, University of Catania, Catania, Italy; 4 Unit of Cranial Posterior Fossa Surgery, University and Hospital Trust of Verona, Verona, Italy; 5 Department of Neurosciences, University of Verona, Verona, Italy

**Keywords:** Rosette-forming glioneuronal tumor, H3 K27-altered diffuse glioma, H3 K27me3, *H3F3A*

## Abstract

According to the fifth edition of the World Health Organization (WHO) classification of tumors of the central nervous system (CNS), diffuse midline glioma H3 K27-altered is a grade 4 infiltrative glioma that arises from midline anatomical structures and is characterized by the loss of H3 K27me3 and co-occurring *H3* K27M mutation or EZHIP overexpression. However, the *H3* K27M mutation has also been observed in circumscribed gliomas and glioneuronal tumors arising in midline anatomical structures, which may result in diagnostic pitfalls.

Rosette-forming glioneuronal tumor (RGNT) is a CNS WHO grade 1 neoplasm that histologically features neurocytic and glial components and originates in midline anatomical structures.

This study aimed to assess whether RGNTs, similar to other midline tumors, may exhibit immunohistochemical loss of H3 K27me3 and harbor the *H3* K27M mutation.

All seven analyzed RGNTs displayed immunohistochemical loss of H3 K27me3 in all tumor cells or H3 K27me3 mosaic immunostaining. In one case, H3 K27me3 loss was associated with the *H3* K27M mutation, whereas the other six cases did not exhibit any *H3* mutations or EZHIP overexpression. During a follow-up period of 23 months, the *H3* K27M-mutant case remained unchanged in size despite partial resection, indicating that the *H3* mutation may not confer higher biological aggressiveness to RGNT.

The immunohistochemical loss of H3 K27me3 co-occurring with the *H3* K27M mutation may result in the potential misdiagnosis of RGNT, especially in cases of small biopsy specimens consisting of only the glial component.

## Introduction

In the fifth edition of the World Health Organization (WHO) classification of central nervous system (CNS) tumors, diffuse midline glioma H3 K27-altered is a pediatric-type diffuse high-grade glioma that arises in midline anatomical structures and displays the immunohistochemical loss of H3 trimethylated in lysine 27 (H3 K27me3), in association with *H3* K27M mutation, or EZHIP overexpression [1]. These tumors preferentially occur in the brainstem, thalamus, or spinal cord and exceptionally in the pineal gland, hypothalamus, and cerebellum [2–5]. Regardless of the presence of histopathological features of malignancy, they are classified as CNS WHO grade 4 [1] because of their poor outcome [6]. However, *H3* K27M mutation has also been reported in other tumors originated in midline anatomical structures and characterized by less aggressive clinical behavior, including pylocytic astrocytomas [7, 8], gangliogliomas [9], glioneuronal tumors not otherwise specified [10], and infratentorial IDH-mutant astrocytomas [11]. The presence of this genetic alteration in tumors other than H3 K27-altered diffuse midline gliomas may result in misdiagnosis. As it was already emphasized by the c-IMPACT-NOW (Consortium to Inform Molecular and Practical Approaches to CNS Tumor Taxonomy—Not Official WHO) [12], the diagnosis of diffuse midline glioma *H3* K27M-mutant should be reserved for gliomas that are infiltrating and originated in midline anatomical structures, and not be extended to other *H3* K27M-mutant tumors [1]. Nevertheless, the immunohistochemical loss of H3 K27me3 or the detection of *H3* K27M mutation may pose diagnostic challenges in small biopsy specimens, where the distinction between circumscribed and diffuse gliomas, or between glial and glioneuronal tumors, may be difficult.

Rosette-forming glioneuronal tumor (RGNT) is a CNS WHO grade 1 biphasic neoplasia that histologically features a component of neurocytic cells arranged in rosettes or perivascular pseudorosettes and a glial component with piloid or oligodendroglia-like cells resembling pylocitic astrocytoma [13]. This rare tumor primarily affects children, adolescents, and young adults, and can involve the fourth ventricle or aqueduct, the brainstem, cerebellar vermis, quadrigeminal plate, pineal gland, or thalamus [13]. RGNT displays a unique DNA methylation profile and distinct genetic features consisting of mutations in the tyrosine kinase domain (either at p.N546 or p.K656) of *FGFR1*, co-occurring mutations of either *PIK3CA* or *PIK3R1*, and in a subset of cases inactivation of *NF1* [14, 15].

In this study, we investigated whether RGNTs, like other midline tumors, may show immunohistochemical loss of H3 K27me3 and harbor the *H3* K27M mutation.

## Materials and methods

### Cases

This study included seven RGNTs obtained from six female and one male patient (aged 18–43 years, with a median age of 29 years). All tumors were localized in midline anatomical structures, including the mesencephalon (*n* = 3), pineal gland (*n* = 2), hypothalamus (*n* = 1), and Sylvian aqueduct (*n* = 1). On imaging, all seven tumors appeared relatively circumscribed; three were described as cystic-solid, whereas four were reported as solid masses (Table 1).

**Table 1:** Clinico-pathological, immunohistochemical, and genetic features of 7 RGNTs studied

Case	Sex	Age	Site	Imaging	Resection	Intra-operative macroscopic description	H3 K27me3 IHC	Histone H3.3 K27M-mutant IHC	EZHIP IHC	Mutations	R (m)
1	F	38	Mesencephalon	Relatively circumscribed; solid	Partial	Soft, well dermarcated, highly vascularized	Lost in >95% cells	Positive	Negative	*FGFR1* (K638R; Y653C; K656Q); *PIK3CA* (E110del); *H3F3A* (K27 M)	No (23)
2	F	22	Pineal gland	Relatively circumscribed; solid	Partial, endoscopic	Soft, well dermarcated, highly vascularized	Lost in >95% cells	Negative	Negative	*FGFR1* (N546K); *PIK3CA* (H1047L); NF1 (L2337R; E2339Dfs*; 2340P); *Top2A* (S1337L); *BLM* (V4A); *ERBB3* (G989V); *NOTCH4* (R1475S); *FGF5* (D106N); *MSH2* (N583I)	No (27)
3	F	29	Mesencephalon	Relatively circumscribed; solid	Partial	Soft, well dermarcated	Lost in >95% cells	Negative	Negative	*FGFR1* (N546K); *PIK3CA* (H1047L); *NF1* (W2317Gfs*2; T2621Lfs*3); *PIK3R1* (Y452del; K575del); *REKQL4* (L719H); *CDKN1B* (S7C); *FGFR4* (M524I); *PAX3* (K183del)	No (134)
4	F	33	Pineal gland	Relatively circumscribed; cystic-solid	Partial, endoscopic	Soft	Mosaic pattern (lost in 40% cells)	Negative	Negative	NA	No (6)
5	M	43	Mesencephalon	Relatively circumscribed; cystic-solid	Partial	Soft, well dermarcated	Mosaic pattern (lost in 60% cells)	Negative	Negative	NA	No (103)
6	F	29	Hypothalamus	Relatively circumscribed; solid	Biopsy	Soft	Mosaic pattern (lost in 40% cells)	Negative	Negative	NA	No (64)
7	F	18	Sylvian aqueduct	Relatively circumscribed; cystic-solid	Gross total	Soft, well dermarcated	Mosaic pattern (lost in 40% cells)	Negative	Negative	NA	No (11)

*R (m)*: recurrence (months). *F*: female. *M*: male. *VAF*: variant allele frequency. *NA*: not assessed

Gross total resection was achieved in only one case, whereas six underwent biopsy or partial resection. No patients had received adjuvant therapy.

Recurrence-free survival (RFS) data were retrieved from the clinical records.

### Immunohistochemistry

Immunohistochemistry was performed in all cases using antibodies against H3 K27me3 (clone C36B11, Cell Signaling Technology, Danvers, MA, USA; dilution 1:200), Histone H3.3 K27M-mutant (polyclonal, Merck KGaA, Darmstadt, Germany; dilution 1:500), and EZHIP/CXorf67 (polyclonal, Merck KGaA, Darmstadt, dilution 1:75), and an automated immunostainer (Leica Biosystems, Newcastle, UK).

H3 K27me3 immunohistochemical expression was considered: (i) lost when immunostaining was absent in >95% neoplastic cells and present in the internal positive controls (endothelium, non-neoplastic cells) [16]; (ii) retained, when nuclear staining was present in >95% of tumor cells; and (iii) mosaic, when the percentage of immunostained neoplastic cells was between 5 and 95%. Histone H3.3 K27M-mutant and EZHIP immuno-expression was classified as present or absent in tumor cells.

### Next-generation sequencing

Cases showing immunohistochemical loss of H3 p.K28me3 and/or Histone H3.3 K27M-mutant immuno-expression were further analyzed using a next-generation sequencing (NGS) panel targeting 523 cancer-relevant genes (TruSight Oncology 500, Illumina, San Diego, CA, USA).

Genomic DNA was extracted from FFPE tissue sections using Maxwell CSC instrument (Promega, Madison, USA) with Maxwell RSC DNA FFPE kit (Promega, Madison, USA) according to the manufacturer’s protocol; DNA concentrations were measured on a Qubit 2.0 Fluorometer (Thermo Fisher Scientific, Waltham, USA) using the Qubit dsDNA High Sensitivity. DNA libraries were prepared using TSO500 Library Preparation Kit (Illumina, San Diego, CA, USA) and sequenced to a mean coverage depth of >500× for up to 500 cancer-related genes. NGS data were analyzed with Illumina TruSight Oncology 500 Local App v2.1 and variant report files were uploaded into the Pierian Clinical Genomics Workspace cloud (Pierian DX software CGW_V6.21.1).

## Results

### RGNTs have lost or mosaic H3 K27me3 immunostaining

**Fig. 1:**
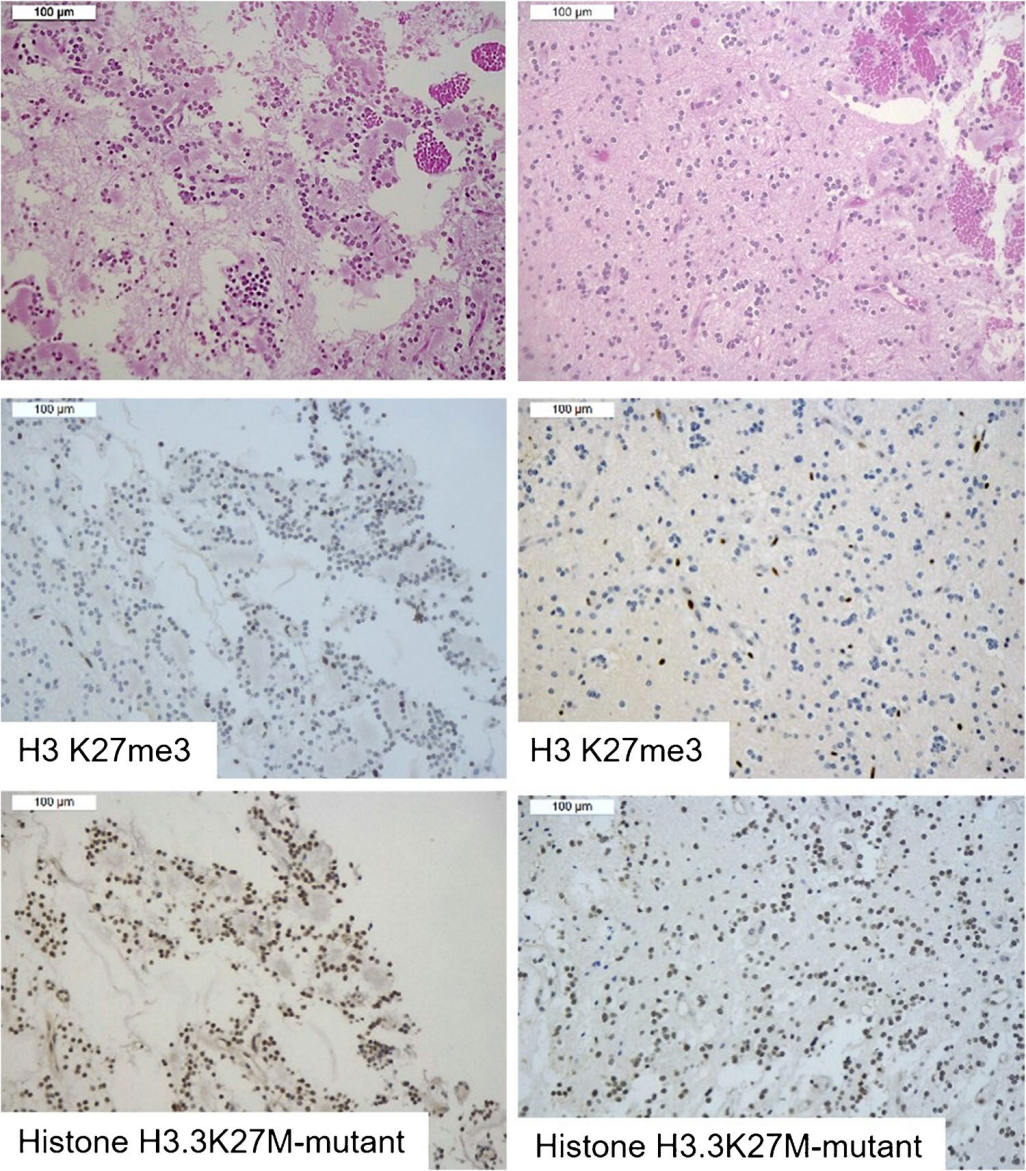
RGNT displaying the immunohistochemical loss of H3K27me3 in the entirety of neoplastic cells, coupled with Histone H3 K27M-mutant positivity (case 1). H3 K27me3 immunohistochemical expression was lost in both the rosette-forming (left) and glial (right) components (endothelial cells retained the immunostaining and served as internal positive control). These latter exhibited Histone H3.3 K27M-mutant immunostaining and were negative for EZHIP. Next-generation sequencing confirmed the presence of *H3.3* K27M mutation

**Fig. 2:**
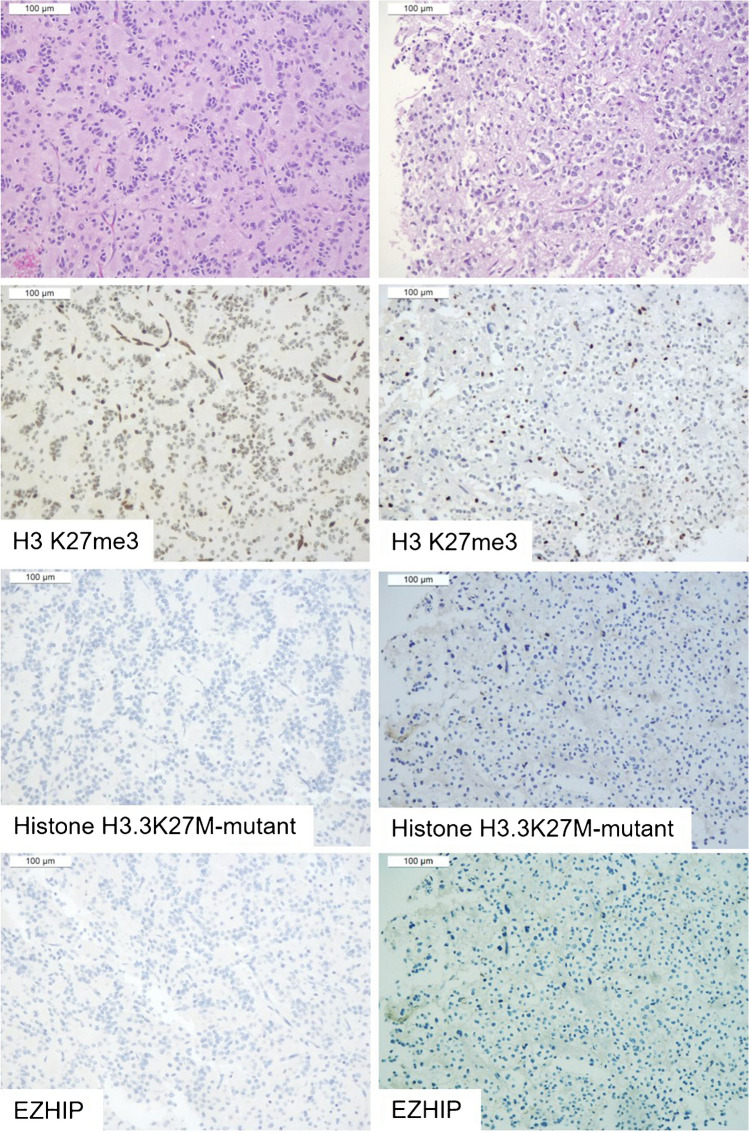
RGNT displaying the immunohistochemical loss of H3K27me3 in the entirety of neoplastic cells, in the absence of any staining for either Histone H3 K27M-mutant or EZHIP (case 2). H3 K27me3 immunohistochemical expression was lost in both the rosette-forming (left) and glial (right) components (endothelial cells retained the immunostaining and served as internal positive control). Both tumor components were negative for either Histone H3.3 K27M-mutant immunostaining or EZHIP. Next-generation sequencing did not reveal *H3F3A*, *HIST1H3B*, and *HIST1H3C* mutations

All RGNTs exhibited loss of H3 K27me3 or mosaic immunostaining. Three cases displayed immunohistochemical loss of H3K27me3 in the entirety of the tumor cells. One of these RGNTs showed positive immunostaining for Histone H3.3 K27M-mutant (case 1) (Fig. 1), whereas the other two cases were negative for both H3.3 K27M-mutant and EZHIP (Fig. 2).

**Fig. 3:**
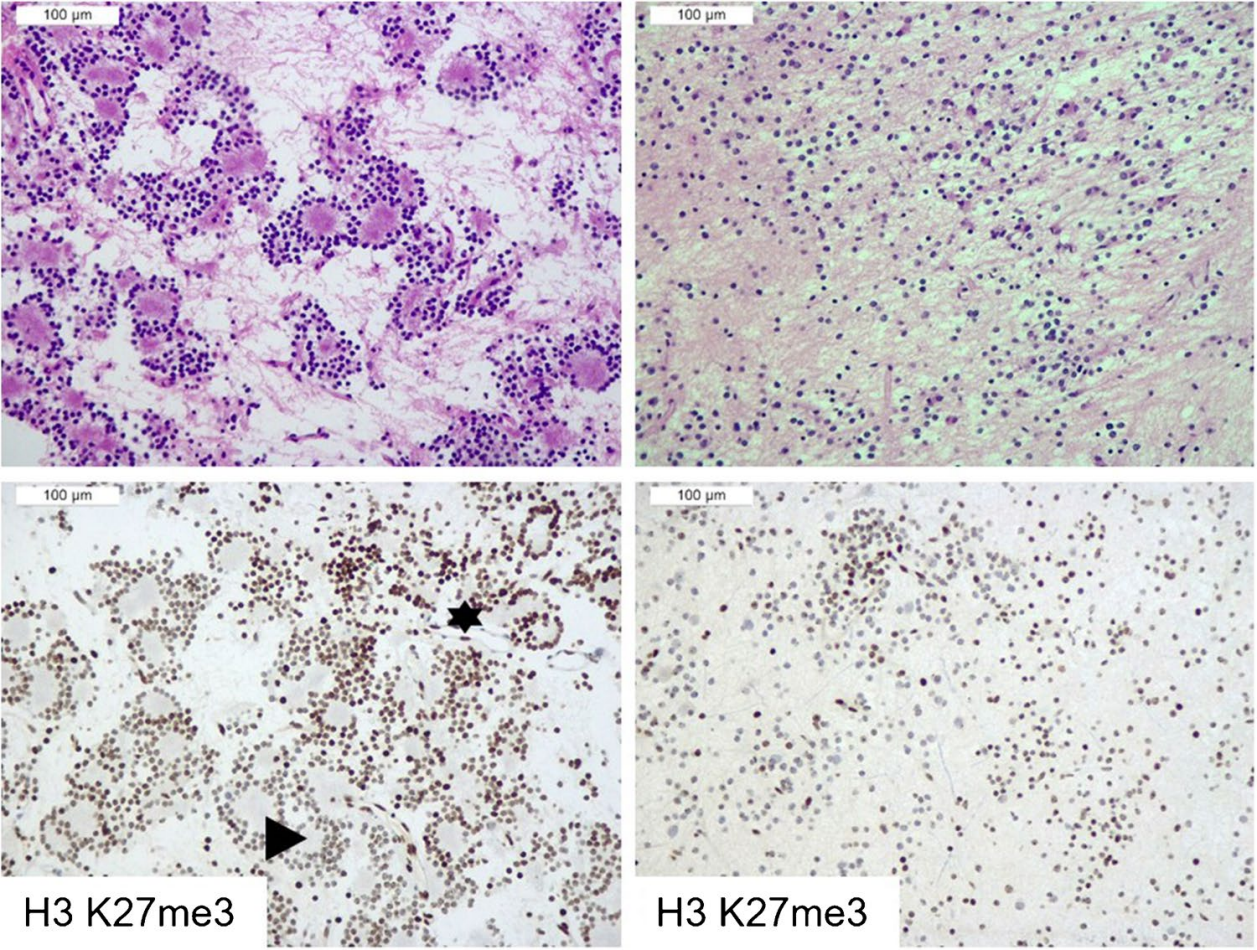
RGNT displaying mosaic immunostaining for H3 K27me3 (case 4). The tumor displayed mosaic expression of H3 K27me3, with alternating positive (star) and negative (triangle) areas in both rosette-forming (left) and glial (right) components

**Fig. 4:**
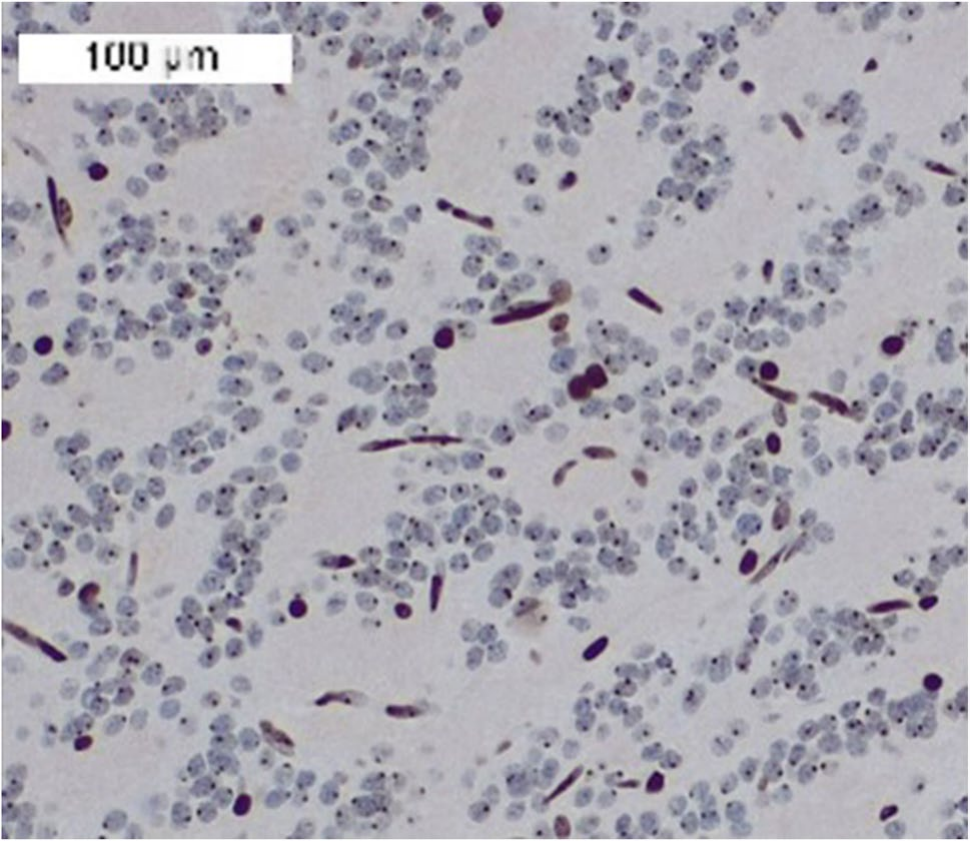
Nuclear dot-like immunostaining for H3 K27me3 in tumor cells of a RGNT in a female patient (case 4). Strong immunostaining in the endothelial cells and nuclear dot-like immunostaining corresponding to inactivated X chromosome

Four RGNTs had mosaic H3 K27me3 expression, with the percentage of neoplastic stained cells ranging between 40 and 60% (Table 1). In the tumor areas with H3 K27me3 loss, endothelial cells displayed strong staining for this protein (Fig. 3). Moreover, in female patients, negative tumor cells exhibited H3 K27me3 dot-like immunostaining in the inactivated X chromosome (Fig. 4). None of RGNTs with H3 K27me3 mosaic expression were positive for H3.3 K27M-mutant or EZHIP.

### H3 K27M mutation may occur in RGNTs

All three RGNTs that showed loss of H3 K27me3 presented with mutations in *FGFR1* at p.N546 or p.K656, and concurrent mutations in *PIK3CA* (Table 1). Of these cases, the one with positive staining for H3.3 K27M-mutant (case 1) harbored the K27M mutation in *H3F3A*, whereas the other two cases did not have any mutations in *H3F3A, HIST1H3B,* and *HIST1H3C.*

The follow-up period for the entire cohort ranged from 5 to 134 months, with a median of 27 months. None of the patients experienced recurrence or re-growth of their tumor. The RGNT with the K27M mutation in *H3F3A* remained stable in size over a 23-month follow-up period (Table 1).

## Discussion

Since the description of diffuse midline glioma *H3* K27M-mutant as a distinct tumor type characterized by a dismal prognosis [17], there have been reports of the *H3* K27M mutation occurring in other tumors. In 2018, a systematic review and meta-analysis of the literature published between 2012 and 2017, conducted by Pratt et al., identified 26 *H3* K27M-mutant circumscribed gliomas originating in midline anatomical structures [18]. The histological diagnoses of these cases included pilocytic astrocytoma, ganglioglioma, anaplastic ganglioglioma, glioneuronal tumor, anaplastic glioneuronal tumor, ganglion cell tumor, anaplastic ependymoma, and circumscribed glioma not otherwise specified [18]. The analysis of survival data, available for 21 cases, showed that patients with circumscribed gliomas *H3* K27M-mutant had longer overall survival compared to patients with both H3 K27-altered and *H3*-wild-type diffuse gliomas, but shorter overall survival than patients with *H3*-wild-type circumscribed gliomas [18]. These results suggest that the *H3* K27M mutation might be a characteristic feature of tumors arising in midline anatomical structures and likely confers higher biological aggressiveness even to circumscribed gliomas compared to cases lacking this genetic alteration [18]. Similarly, the *H3* K27M mutation was associated with worse prognosis in infratentorial *IDH*-mutant astrocytomas [11]. However, due to reports of pilocytic astrocytomas, glioneuronal tumors, or subependymomas with a favorable prognosis despite the *H3* K27M mutation [8–10, 19], the prognostic significance of the *H3* K27M mutation in tumors other than diffuse gliomas remains unclear.

To the best of our knowledge, this is the first study to report a *H3* K27M mutation in RGNT. In this particular case, the tumor cells showed positive immunostaining for the Histone H3.3 K27M-mutant and a concordant immunohistochemical loss of H3 K27me3. The *H3* K27M mutation was confirmed by NGS, which also identified three mutations in the tyrosine kinase domain of *FGFR1* (amino acids 478–767) and a mutation in *PIK3CA*. Over the course of 23 months, the tumor has demonstrated no evidence of regrowth, and has remained unchanged in size. This suggests that RGNTs with the *H3* K27M mutation do not exhibit biological behavior consistent with that of high-grade tumors.

Another novel finding of this study was that all seven analyzed RGNTs exhibited a loss of immunohistochemical H3 K27me3 in at least a proportion of cells, with three cases displaying the absence of H3 K27me3 in the entirety of the tumor cells. A previous study analyzed the pattern of H3 K27me3 immunostaining in two RGNTs and both retained the expression of this protein, although the percentage of positive cells was not detailed [20]. Notably, the RGNTs with mosaic expression displayed strong H3K27me3 immunostaining in the endothelial cells within the areas with loss of staining in the neoplastic cells, thus ruling out a technical artifact. A similar pattern of H3 K27me3 immunostaining was reported in 22 out of 62 (35%) atypical meningiomas [21]. Further substantiating that this staining pattern is not artifactual, in tumors from female patients, the negative tumor cells displayed H3 K27me3 dot-like immunostaining in the inactivated X chromosome, as previously described in diffuse gliomas [22].

The loss of trimethylation of lysine 27 in the H3 protein may result from the *H3* K27M mutation, which hinders the biochemical inhibition of the Polycomb Repressor Complex 2 (PRC2) [6]. Alternatively, it may originate from EZHIP overexpression, which inhibits the functional enzymatic component EZH2 of PRC2 [23]. However, excluding the case harboring the K27M mutation in *H3F3A*, none of the other six RGNTs had mutations in histone genes or EZHIP overexpression, suggesting that H3 K27me3 loss may be attributed to other mechanisms that impede PRC2 function.

H3 K27me3 immunohistochemical loss has been observed in various CNS tumors, regardless of their location in midline anatomical structures. This loss of H3 K27me3 expression has been reported to predict a poor prognosis in ependymomas occurring in the posterior fossa [24] as well as a shorter time to recurrence and resistance to radiosurgery in meningiomas [25]. However, in diffuse *IDH*-mutant hemispheric gliomas, the immunohistochemical loss of H3 K27me3 is associated with a significantly better prognosis [16]. In this study, none of the RGNTs experienced regrowth or recurrence over a range of 5 to 134 months. Although some cases had limited follow-up time, these findings suggest that H3 K27me3 loss has no prognostic significance in RGNTs. Recently, Kim et al. have reported that H3 K27me3 immunoexpression was lost in all 25 central neurocytomas and three pituicytomas analyzed in absence of *H3F3A* mutations and EZHIP overexpression [26]. Therefore, the spectrum of tumors with loss of trimethylation at lysine 27 of H3 is broader than initially believed and encompasses diverse tumor types arising from midline structures through different pathogeneses.

In conclusion, this study is the first to demonstrate that the *H3* K27M mutation and immunohistochemical loss of H3 K27me3 may characterize RGNTs. This should be acknowledged to avoid misdiagnosis as diffuse midline glioma H3 K27-altered, especially in the case of small biopsy specimens of RGNT showing the sole glial component of the tumor, and where the absence of infiltrating features may not be easily discernible. In these cases, radiological or intra-operative impression of a well-circumscribed tumor may be helpful in the differential diagnosis. Indeed, all seven cases in this cohort appeared to be relatively circumscribed on imaging, as previously reported in other series [27], and five were described as well-demarcated in the surgery reports as well. In addition, DNA methylation profiling or genetic analyses involving *FGFR1*, *PIK3CA*, and *PIK3R1* may aid in resolving the differential diagnosis of H3 K27-altered diffuse gliomas. Indeed, co-occurring mutations in *FGFR1* and *PIK3CA* or *PIK3R1* were evidenced in the majority of RGNTs [14, 15, 28]. On the other hand, the co-occurrence of these mutations was exceptionally observed in H3 K27-altered diffuse gliomas [29–31].

## Data Availability

Data will be available upon request to corresponding author.
